# Overdiagnosis Due to Screening Mammography for Breast Cancer among Women Aged 40 Years and Over: A Systematic Review and Meta-Analysis

**DOI:** 10.3390/jpm13030523

**Published:** 2023-03-14

**Authors:** Arwa F. Flemban

**Affiliations:** Pathology Department, Faculty of Medicine, Umm Al-Qura University, Makkah 21955, Saudi Arabia; aflemban@uqu.edu.sa; Tel.: +966-549300940

**Keywords:** breast cancer, mammography, meta-analysis, overdiagnosis, screening

## Abstract

The current systematic review and meta-analysis was conducted to estimate the incidence of overdiagnosis due to screening mammography for breast cancer among women aged 40 years and older. A PRISMA systematic search appraisal and meta-analysis were conducted. A systematic literature search of English publications in PubMed, Web of Science, EMBASE, Scopus, and Google Scholar was conducted without regard to the region or time period. Generic, methodological, and statistical data were extracted from the eligible studies. A meta-analysis was completed by utilizing comprehensive meta-analysis software. The effect size estimates were calculated using the fail-safe N test. The funnel plot and the Begg and Mazumdar rank correlation tests were employed to find any potential bias among the included articles. The strength of the association between two variables was assessed using Kendall’s tau. Heterogeneity was measured using the I-squared (I2) test. The literature search in the five databases yielded a total of 4214 studies. Of those, 30 articles were included in the final analysis, with sample sizes ranging from 451 to 1,429,890 women. The vast majority of the articles were retrospective cohort designs (24 articles). The age of the recruited women ranged between 40 and 89 years old. The incidence of overdiagnosis due to screening mammography for breast cancer among women aged 40 years and older was 12.6%. There was high heterogeneity among the study articles (I2 = 99.993), and the pooled event rate was 0.126 (95% CI: 15 0.101–0.156). Despite the random-effects meta-analysis showing a high degree of heterogeneity among the articles, the screening tests have to allow for a certain degree of overdiagnosis (12.6%) due to screening mammography for breast cancer among women aged 40 years and older. Furthermore, efforts should be directed toward controlling and minimizing the harmful consequences associated with breast cancer screening.

## 1. Introduction

Breast cancer is the most common invasive cancer in women globally [1]. Breast cancer was the most frequently diagnosed malignancy in 2020. The World Health Organization (WHO) stated that 2.3 million new cases were diagnosed and it resulted in 685,000 fatalities worldwide in 2020 [2]. The WHO also reported that, as of the end of 2020, there were 7.8 million women alive who had been diagnosed with breast cancer in the past 5 years, making it the world’s most prevalent cancer [3]. Breast cancer incidence is closely correlated with an increase in age [4]. Only 5% of all breast cancers occur in women under 40 [5]. Early identification and treatment of breast cancer are effective in high-income nations, and there is a limited benefit of screening in low-income countries due to expenses [6].

Mammography and the early detection of breast cancer save the lives of two out of three women, highlighting the benefit of mammography screening. A vast amount of published work highlights the benefit of early diagnosis of breast cancer via mammography screening. In the late 20th century, screening mammography was implemented in high-income nations, based on the finding that it decreased the mortality rate from breast cancer, without proper consideration or knowledge of potential risks [7]. Additionally, mammography screening programs started before the development of hormonal therapy and other targeted therapies for breast cancer. Thus, early mammography randomized control trial results showed poor adherence to the principles of screening set by the World Health Organization [8]. This sparked a debate in research about the benefits and drawbacks of mammography-based breast cancer screening [9,10].

Several risks are associated with breast cancer screening with mammography, including overdiagnosis and false-positive results. With the increase in breast cancer awareness and the success of breast cancer early detection campaigns, the number of women enrolling for mammography has increased significantly. False-positive cases are usually detected with confirmation and are not subjected to further treatment, however, this results in additional costs for confirmatory tests and required procedures. On the other hand, the risk of overdiagnosis is difficult to determine [11].

Over more than 15 years, there has been increasing awareness of the overdiagnosis of breast cancer. Cancer overdiagnosis is the detection of tumors that would not become symptomatic, nor would they have progressed to life-threatening disease during the life of the patient being screened [12]. Overdiagnosis refers to a screen-detected malignancy that would not have progressed to clinical or symptomatic presentation during the individual’s lifetime and would not have been diagnosed nor caused the individual any harm in the absence of screening. This somewhat contested harm of cancer screening, one that is inherently difficult to quantify, adds to the complexity of the outcomes associated with mammography screening [13]. Overdiagnosis is currently a growing ethical dilemma due to the burdens it places on patients and healthcare systems, including physical, labor, and financial strains [14]. Almost all cancer patients are provided with therapy since it is currently impossible to determine whether patients would benefit or suffer harm from early detection and treatment. So, overtreatment of overdiagnosed cases can increase mortality rates with no beneficial outcomes [15]. Overdiagnosed cases are subjected to overtreatment, unnecessary surgery, radiotherapy, and other adjuvant therapy. This overtreatment does not benefit patients and may cause undue harm in the form of physical and psychological effects that lower quality of life and shorten life expectancy [15]. There is another layer of complexity added to the topic of overdiagnosis: the difficulty of producing a precise estimate of its magnitude in newly diagnosed cases. Therefore, it is crucial for science and public health to determine the prevalence and frequency of overdiagnosis.

Overdiagnosis is an unexpected but inevitable danger when trying to detect pre-symptomatic cancer in age groups at considerable risk of death from other causes. However, early detection of breast cancer may result in a decrease in mortality [16]. There has been no universally accepted method to measure the value of the overdiagnosis of breast cancer [17]. The first hurdle in estimating the value of overdiagnosis involves the nature and subtype of breast cancer. Breast cancer is a heterogeneous disease and involves both in-situ and invasive subtypes. On initial thought, in-situ cancers’ progression to invasive cancers takes longer and, thus, results in a longer lead time [18]. This has led to the notion that there could be a higher risk of overdiagnosis for in-situ tumors compared to invasive ones; however, this notion was proven false, as the nature of tumors includes high genetic and inherited diversity [18]. This is why there has been a high level of variation in reported overdiagnosis in previous studies, ranging from 0 to 54% [19,20]. The studies that utilized lead time adjustment found that overdiagnosis was as low as 5%, while the observational studies reported overdiagnosis rates as high as 54% [19]. Another level of complexity is added because some forms of invasive carcinoma are non-progressive and can regress in the patient’s lifetime. This has led several studies to include the growth rate of tumors in determining overdiagnosis, which has resulted in an underestimation of the value of overdiagnosis [21].

The statistics and evidence from published work have reported variations in both the decreases in mortality and overdiagnosis using mammography [17]. Additionally, the magnitude of collective harm resulting from overdiagnosis does not essentially outweigh the benefit of breast cancer screening. Thus, many studies have recommended the planning of modifications to screening programs to reach a proper balance of benefits with minimal levels of harm [17]. These plans should be based on correctly assessing the level of overdiagnosis that causes unwanted harm while simultaneously measuring the benefit of screening in terms of mortality reduction. Further, the complete disclosure of these data to participants in the screening program should occur to make sure that they are aware of all of the potential benefits as well as harms associated with screening.

Furthermore, based on this evidence, more effort should be put into studying approaches to reducing the overtreatment of breast cancer-detected cases [22]. Thus, this systematic review and meta-analysis aim to fill this evidence gap. Women who want to be screened must accept an additional risk of diagnosis and treatment, including the risk of overdiagnosis and overtreatment, if they want to lower their risk of dying from breast cancer [23]. However, establishing the incidence and frequency of overdiagnosis is critically important to determine whether the net benefit justifies the resources required for screening and to provide the best information possible to help healthy women weigh the potential benefits versus the potential harms of participating in breast cancer screening. Therefore, the current systematic review and meta-analysis were conducted to estimate the incidence of overdiagnosis due to screening mammography for breast cancer among women aged 40 years and over.

## 2. Materials and Methods

### 2.1. PRISMA Guidelines and Protocol Registration

The Preferred Reporting Items for Systematic Reviews and Meta-Analyses (PRISMA) criteria were followed in the creation of this systematic review and meta-analysis (Table S1). The International Prospective Register of Systematic Reviews received the study protocol for registration (PROSPERO, registration No. CRD42022383242).

### 2.2. Literature Search Strategy

Without regard to the region or time period, the author conducted a thorough and methodical search of English publications published in PubMed, Web of Science, EMBASE, Scopus, and Google Scholar. A mixture of search techniques was used to broaden the scope of the search: the first was a MESH (medical subject header) search using the terms “Overdiagnosis”, “Mammography”, and “Breast Cancer”; the second was a free-text search using the following phrases (Women, Female, Breast Neoplasms, Incidence, Trend, Breast Neoplasms/epidemiology, Mass Screening, Mammography, Screening, and Overdiagnosis). Synonyms were combined using the boolean operator (OR), and cases and tests were combined using the boolean operator (AND) (Table S2).

### 2.3. Eligibility Criteria (Inclusion/Exclusion)

Articles examined the incidence of overdiagnosis due to screening mammography for breast cancer among women aged 40 years and over were included in this systematic review and meta-analysis. However, review articles, case report articles, articles written in languages other than English, articles deficient in relevant information, and articles without the full text were excluded.

### 2.4. Study Screening

The EndNote V.X8 software was deployed for the management of the article screening process. The duplicates were deleted, and then, the author methodically chose the included articles by screening the titles, abstracts, and full texts of the publications.

### 2.5. Data Extraction

The necessary information was gathered into a standardized table with the following headings: first author name and year of publication, study setting, study design, study participants, sample size, type of mammography, screening period, screening interval, number of positive screenings, and calculation of the overdiagnosis rates (%). Additionally, to improve accuracy and critical appraisal, data extraction was conducted by three independent researchers, and disputes between researchers were resolved through consensus.

### 2.6. Quality Assessment

The effectiveness of the included studies was evaluated using the Quality Assessment Tool for Quantitative Studies (QATFQS), which was developed by the Effective Public Health Practice Project (EPHPP) [24]. Each object was subjected to eight tool questions, which were individually graded with a “1” signifying excellent quality, a “2” signifying good quality, and a “3” signifying bad quality. The next step was to determine the overall ranking for each study using the following criteria: “1” indicates good quality (no poor ratings), “2” indicates moderate quality (one weak rating), and “3” indicates mediocre quality (two or more weak ratings) [25].

### 2.7. Meta-Analysis

The meta-analysis was completed using Comprehensive Meta-Analysis Software (CMA, version 3, BioStat, Tampa, FL, USA) (CMA, version 3, BioStat, Tampa, FL, USA). The effect size estimates of the included studies were calculated using the fail-safe N test.

The funnel plot was used to identify potential publication bias, and the Begg and Mazumdar rank correlation test was employed to find any potential bias among the included publications. The strength of the association between two variables was assessed using Kendall’s tau.

The heterogeneity between the included articles was measured using the I-squared (I2) statistic, and values of 25%, 50%, and 75% were classified as low, moderate, and high estimates, respectively [26]. When the *p*-value is more than 0.05, statistical heterogeneity is believed to be non-significant. The high degree of variability served as the impetus for the adoption of a random-effects model [27].

## 3. Results

### 3.1. Search Findings

**Figure 1:**
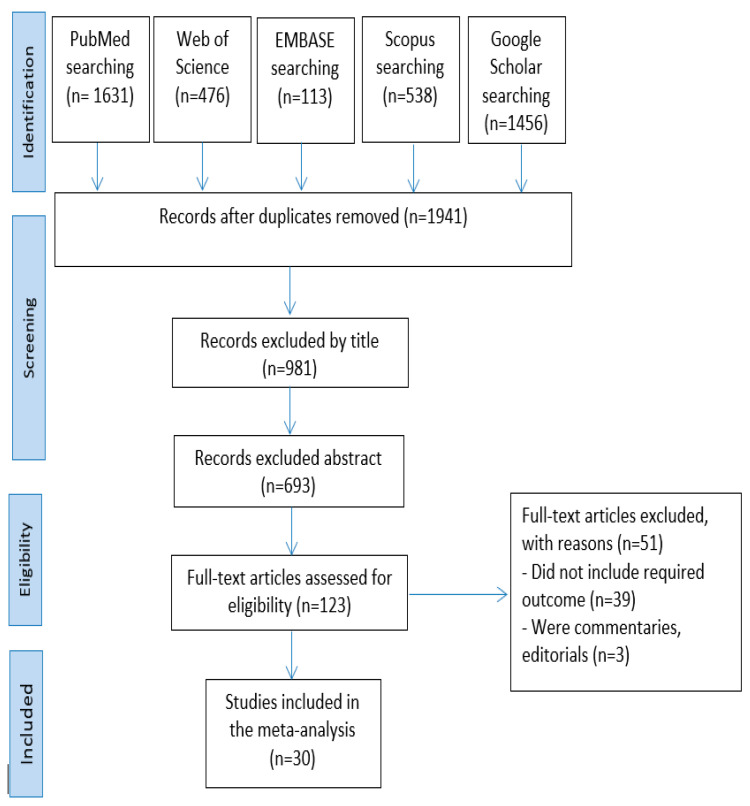
Process for choosing articles.

The search in the five databases yielded a total of 4214 articles: 1631 in PubMed, 467 in Web of Science, 113 in EMBASE, 538 in Scopus, and 1456 in Google Scholar. After eliminating the duplicate entries, 1941 articles were left. Then, after filtering the titles and abstracts, 960 and 288 articles, respectively, were eliminated. The remaining 123 articles’ full texts were evaluated. Of these, 93 items were ultimately disqualified for not fitting the inclusion requirements. In the end, 30 articles were chosen for qualitative synthesis and meta-analysis after the screening procedure (Figure 1).

### 3.2. Characteristics of the Included Articles

The first study on the incidence of overdiagnosis due to screening mammography for breast cancer among women aged 40 years and over was published in 2004; two articles were published in 2006, one article in 2009, one article in 2010, two articles in 2011, three articles in 2012, three articles in 2013, one article in 2014, two articles in 2015, five articles in 2016, three articles in 2017, two articles in 2019, one article in 2020, one article in 2021, and two articles in 2022. Most studies were conducted in Norway (four) and the United Kingdom (four), followed by the United States (three), France (three), Denmark (three), Australia (three), the Netherlands (two), Belgium (one), Italy (one), Taiwan (one), Canada (one), Sweden (one), and Finland (one). The vast majority of the articles employed a retrospective cohort design (24 articles). The age of the recruited women in the included articles ranged between 40 and 89 years old. The sample size of the included articles ranged between 451 and 1,429,890 women, with an average of 237,019 women.

All the included articles reported that the type of mammography was biennial two-view mammography. The screening interval in the included articles ranged between 2 and 40 years, with an average of 15.1 years. The calculation of overdiagnosis (%) in the included articles ranged between 0.7% and 52% years, with an average of 16.6% (Table 1).

### 3.3. Unified Findings

The effect analysis of the 30 included articles in the current meta-analysis showed that the points of the estimate of the incidence of overdiagnosis due to screening mammography for breast cancer among women aged 40 years and over were 0.177 and 0.126 according to the fixed and random models, respectively. The Q-value, calculated using the homogeneity test, showed that the incidence of overdiagnosis due to screening mammography for breast cancer among women aged 40 years and over has a heterogenous structure (Q = 2137.239; *p* < 0.001) and (Q = 413,510.290; *p*-value < 0.001). As a result, the author completed the current meta-analysis using the random-effects model in order to lessen the misunderstandings that the discrepancy of the articles caused. The tau value was 0.480, which represents the true overall heterogeneity between the included articles.

A high level of heterogeneity was obtained, as indicated by the I-squared (I2) value of 99.993, thus indicating that the random-effects model for meta-analysis should be applied (Table 2).

### 3.4. The Incidence of Overdiagnosis Due to Screening Mammography for Breast Cancer among Women Aged 40 Years and Over

**Figure 2:**
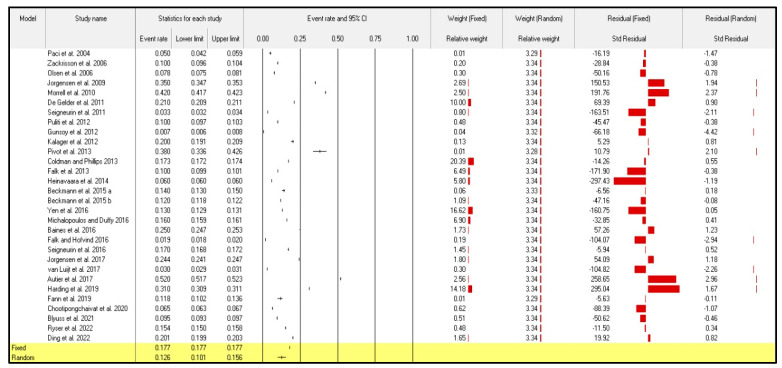
Forest plot from the fixed- and random-effects analyses: the incidence of overdiagnosis due to screening mammography for breast cancer among women aged 40 years and over [21,22,23,24,25,26,27,28,29,30,31,32,33,34,35,36,37,38,39,40,41,42,43,44,45,46,47,48,49,50].

Despite the random-effects meta-analysis showing a high degree of heterogeneity among articles, I2 = 99.993, the pooled event rate (and 95% CI) was 0.126 (95%CI: 0.101–0.156) (Figure 2). Results indicated that the incidence of overdiagnosis due to screening mammography for breast cancer among women aged 40 years and over was 12.6%.

### 3.5. Fail-Safe N Method

By estimating how many studies with effect sizes of zero might be included in the meta-analysis before the result lost statistical significance, the fail-safe N method was used to evaluate the robustness of a significant finding. The Z-value for the observed studies was 1200.30110, indicating that publication bias could have affected the effect value that our meta-analysis produced (Table 3).

### 3.6. Rank Correlation

The Begg and Mazumdar test showed a weak negative association between the incidence of overdiagnosis due to screening mammography for breast cancer among women aged 40 years and over. The tau value, with and without continuity correction, was −0.10345. Furthermore, there was a weak negative association between the incidence of overdiagnosis due to screening mammography for breast cancer among women aged 40 years and over, where the tau value, with and without continuity correction, was −0.10115. Egger’s test for a regression intercept provided a *p*-value of 0.20746 for the incidence of overdiagnosis due to screening mammography for breast cancer among women aged 40 years and over, indicating the presence of publication bias (Table 4).

### 3.7. Publication Bias

**Figure 3:**
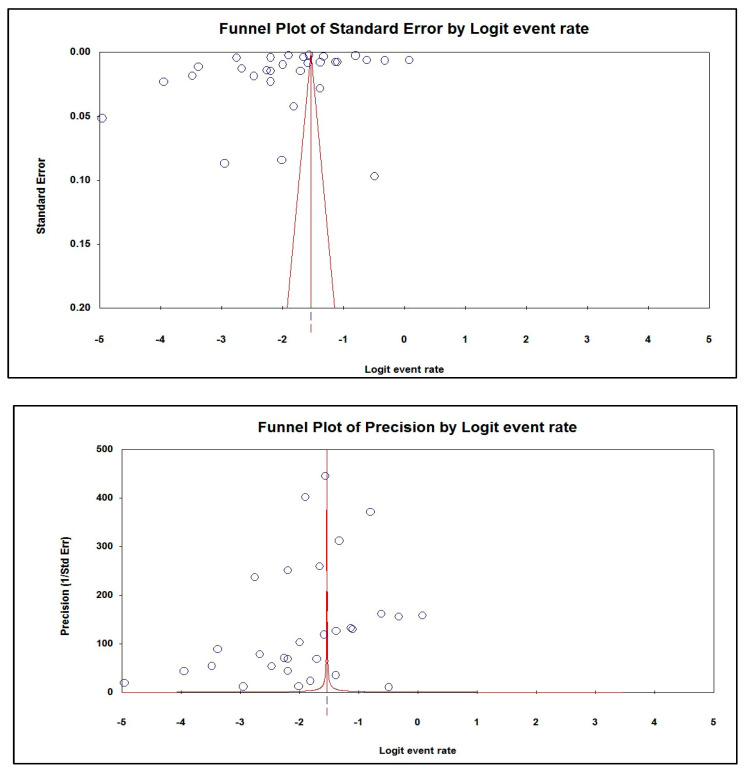
Publication bias of the incidence of overdiagnosis due to screening mammography for breast cancer among women aged 40 years and over.

The asymmetric funnel plots of the incidence of overdiagnosis due to screening mammography for breast cancer among women aged 40 years and over suggested publication bias (Figure 3).

## 4. Discussion

Mammography screening has benefits and drawbacks. Screening mammography lowers a woman’s chance of dying from breast cancer through the early detection of tumors while they are treatable and manageable. Yet, the benefit of mammography screening must be considered against the danger of unwarranted diagnosis and overtreatment that leads to physical, psychological, and financial harm, including the psychological and behavioral effects of labeling; the consequences of subsequent testing (including invasive tests), treatment, and follow-up; and the financial effects on the individual who is overdiagnosed and on society [58]. Additionally, according to the WHO regulations, screening invitations must be accompanied by accurate information about the advantages, risks, and uncertainties of screening mammography [59,60,61]. All relevant information regarding breast cancer mortality reduction due to treatment following early diagnosis and accurate statistics of the rates of overdiagnosis by mammography should be presented to women to help them make well-informed decisions [62].

The estimation of the level of overdiagnosis of breast cancer by screening mammography has been a hot topic in an era of emphasis on enhancing cost-effectiveness and achieving reductions in harm. Ong et al. (2015) showed that, in the United States (US), there were high costs resulting from false-positive mammograms and breast cancer overdiagnosis among women ages 40–59, as based on expenditure data from a major US healthcare insurance plan provider for 702,154 women in the years 2011–2013. The average expenditures for each false-positive mammogram, invasive breast cancer, and ductal carcinoma in situ in the 12 months following diagnosis were $852, $51,837, and $12,369, respectively. This translates to a national cost of $4 billion each year. They concluded that the costs associated with false-positive mammograms and breast cancer overdiagnosis appear to be much higher than previously documented. Screening has the potential to save lives. However, the economic impact of false-positive mammography results and breast cancer overdiagnosis must be considered in the debate about the appropriate populations for screening [63].

Biesheuvel and colleagues reported one of the earliest systematic reviews of breast cancer overdiagnosis and noted that source (primary) studies were prone to biases that may over- or under-estimate the magnitude of breast cancer overdiagnosis. They reported an extremely wide range of overdiagnosis estimates (from 0 to 62%) [64]. Therefore, the current systematic review and meta-analysis were conducted to estimate the incidence of overdiagnosis due to screening mammography for breast cancer among women aged 40 years and over. In the current systematic review and meta-analysis, a search of the five databases yielded a total of 4214 articles. Of these, 30 articles were included in the final analysis, with sample sizes ranging from 451 to 1,429,890 women and including an average of 237,019 women. The articles on the incidence of overdiagnosis due to screening mammography for breast cancer among women aged 40 years and over were published between 2004 and 2022. The age of the recruited women in the included articles ranged between 40 and 89 years old. Furthermore, most of the articles (24 articles) were retrospective cohort studies. Moreover, all the included articles reported that the type of mammography was biennial two-view mammography. Additionally, the screening interval in the included articles ranged between 2 and 40 years, with an average of 15.1 years.

The main results of the current systematic review and meta-analysis indicate that the incidence of overdiagnosis due to screening mammography for breast cancer among women aged 40 years and over was 12.6%. Evidence from observational studies and randomized controlled trials demonstrates that mammography screening lowers the chance of dying from breast cancer. These studies also provide substantial proof that overdiagnosis is a severe problem resulting from community breast screening [65,66,67]. According to the random-effects meta-analysis, there were high degrees of heterogeneity among the included articles [63]. The International Agency for Research on Cancer (IARC) Working Group noted that there was sufficient evidence of overdiagnosis [68]. The Euroscreen Group’s summary emphasized that the estimate of overdiagnosis was 6.5% (ranging from 1% to 10%), based on a systematic review of European studies, and they also incorporated a lead time adjustment [69,70]. This study’s conclusion was based on a systematic evaluation of studies conducted in Europe, and one of the main outcomes of this study was the lead time adjustment [71].

When a malignancy is overdiagnosed, it is one that would not have progressed to clinical or symptomatic manifestation during the patient’s lifetime, would not have been diagnosed, and would not have harmed the patient [72]. Overdiagnosis is difficult to quantify, and outcomes of mammography screening are complicated by this hotly debated harm of cancer screening. A variety of factors add to the complexity of quantifying overdiagnosis. These factors include the mean measurement of the definition of overdiagnosis (which could be presented as the rate or proportion being measured) and the denominator (which is measured either by the number of screened women in long-term follow-up or as a proportion of the cases diagnosed during the screening phase). These considerations contributed to the variability in the reported estimates of breast cancer overdiagnosis attributed to mammography screening. Another factor that adds to the complexity of determining mammography overdiagnosis is factoring in the heterogeneous nature of breast (ductal carcinoma in situ (DCIS)) or invasive cancer (or both). Additionally, the timing of measuring overdiagnosis and the length of follow-up following screening are important factors to add into consideration. Moreover, differences in study populations, including demographics and differences in underlying breast cancer risk, are further considerations. The effect of basic screening methodology is an important factor in measuring overdiagnosis. Other variations in screening practices, including the screening technology used, screening policy and frequency, population coverage, and uptake, are factors that should be considered in the estimation of the risk of overdiagnosis. Furthermore, statistical techniques, adjustments, and assumptions relating to lead time and disease progression (the latter of which is not limited to modeling studies); and the framing of the extent of overdiagnosis (relative or absolute estimates) should be clearly considered in overdiagnosis analysis studies [43,70,73].

Although overdiagnosis estimates are associated with a high degree of uncertainty, estimates of magnitude for the two outcomes of screening—the reduction in breast cancer mortality and overdiagnosis—differ across studies. Though the balance between the advantages and disadvantages of breast cancer screening, including overdiagnosis, is more delicate than first thought, the evidence offered in the overdiagnosis section does not disprove the value of breast cancer screening. Future efforts should be focused on ensuring that any changes in the implementation of breast cancer screening optimize the balance between benefits and harms, including assessing how planned and actual changes modify the risk of overdiagnosis. Further, women should be provided with well-calculated statistics and balanced information about the outcomes that may affect them when participating in screening. Additionally, researchers should focus on reducing the risk of ever-treating detected cases in screening programs.

One limitation of this systematic review and meta-analysis was the high heterogeneity among studies articles. This meta-analysis is the first study that shows the incidence of overdiagnosis due to screening mammography for breast cancer among women aged 40 years and over. The present meta-analysis study could be a data baseline for the incidence of overdiagnosis due to screening mammography for breast cancer among women and can guide other researchers to design new studies.

## 5. Conclusions

Despite the random-effects meta-analysis showing a high degree of heterogeneity among articles, the screening tests have to allow for a certain degree of overdiagnosis (12.6%) resulting from screening mammography for breast cancer among women aged 40 years and older. The magnitude of breast cancer overdiagnosis attributed to mammography screening is complicated by the heterogeneity of many of the elements, political and scientific, that define and interpret the evidence of this screening harm. There is sufficient evidence to acknowledge overdiagnosis as a serious harm caused by breast cancer screening. Based on the available evidence, it is reasonable to conclude that mammography screening reduces the risk of breast cancer death; however, the harms, including overdiagnosis, should be balanced. The snapshot of evidence presented on the incidence of overdiagnosis in this systematic review and meta-analysis, however, does not mean that population breast screening is worthless, given that screening reduces breast cancer deaths. Hence, efforts should be directed toward controlling and minimizing the harmful consequences associated with breast cancer screening. The changes in the implementation of mammography screening plans should ensure a balance between benefits and harms. Full disclosure to the participating women, of all of the outcomes that may affect them when they participate in screening, is necessary as well. This should be sublimated by changes in the regulation of management and treatment of diagnosed cases during screening to minimize overtreatment, thereby reducing the harm of overdiagnosis.

## Figures and Tables

**Table 1:** The data extracted from the included articles.

First Author Name and Year of Publication	Study Setting	Study Design	Study Participants	Sample Size	Type of Mammography	Screening Period	Screening Interval (Years)	Positive Screening	Calculation of Overdiagnosis (%)
Paci et al., 2004 [28]	Italy	Retrospective cohort	Women aged 50–69 years	2780	Biennial two-view mammography	1990–1999	9	2626 invasive cancers and 154 other cancers	5.0
Zackrisson et al., 2006 [29]	Sweden	Randomized screening trial	Women aged 45–69 years	1,711,690	Biennial two-view mammography	1976–1986	10	5050 breast cancer cases	10.0
Olsen et al., 2006 [30]	Denmark	Retrospective cohort	Women aged 50–69 years	40,000	Biennial two-view mammography	1943–1977	34	329 invasive cancers and 50 other cancers	7.8
Jorgensen et al., 2009 [31]	Denmark	Retrospective cohort	Women aged 50–69 years	115,270	Biennial two-view mammography	1991–2003	13	5189 breast cancer cases	35.0
Morrell et al., 2010 [32]	Australia	Retrospective cohort	Women aged 50–69 years	100,000	Biennial two-view mammography	1999–2001	2	830 breast cancer cases	42.0
De Gelder et al., 2011 [33]	Netherlands	Retrospective cohort	Women aged 49–74 years	586,550	Biennial two-view mammography	2004–2006	2	4546 invasive cancers	21.0
Seigneurin et al., 2011 [34]	France	Retrospective cohort	Women aged 50–69 years	245,000	Biennial two-view mammography	1991–2006	15	3675 invasive cancers and 68,600 carcinomas in-situ	3.3
Puliti et al., 2012 [35]	Italy	Retrospective cohort	Women aged 50–69 years	52,282	Biennial two-view mammography	1991–1993	2	1583 breast cancer caces	10.0
Gunsoy et al., 2012 [36]	United Kingdom	Randomized controlled trial	Women aged 40–49 years	53,890	Biennial two-view mammography	1991–2010	19	151 invasive carcinomas and 43 in-situ carcinomas	0.7
Kalager et al., 2012 [37]	Norway	Retrospective cohort	Women aged 50–69 years	7793	Biennial two-view mammography	1985–2005	19	7793 invasive breasts cancers	20.0
Pivot et al., 2013 [38]	France	Cross-sectional	Women aged 40–75 years	451	Biennial two-view mammography	18–30 January 2013	12	NM	38.0
Coldman and Phillips 2013 [39]	United Kingdom	Retrospective cohort	Women aged 40–89 years	1,387,197	Biennial two-view mammography	1970–2009	39	74,189 invasive breasts cancers and 8286 ductal carcinomas in-situ	17.3
Falk et al., 2013 [40]	Norway	Retrospective cohort	Women aged 50–69 years	702,131	Biennial two-view mammography	1995–2009	14	94,085 invasive breast cancer tumors and 43,532 ductal carcinoma in-situ	10.0
Heinavaara et al., 2014 [41]	Finland	Retrospective cohort	Women aged 50–59 years	45–69	Biennial two-view mammography	1935–1939	4	2583 invasive breast carcinomas and 117 other breast carcinomas	6.0
Beckmann et al., 2015 [42]	Australia	Case-control	Women aged 45–85 years	25,373	Biennial two-view mammography	2006–2010	4	4088 invasive breast cancer tumors and 495 ductal carcinoma in-situ	14.0
Beckmann et al., 2015 [43]	Australia	Retrospective cohort	Women aged 40–84 years	100,000	Biennial two-view mammography	1989–2009	20	8611 invasive breast cancers	12.0
Yen et al., 2016 [44]	Taiwan	Retrospective cohort	Women aged 40–69 years	1,429,890	Biennial two-view mammography	1999–2009	10	4423 breast cancer cases	13.0
Michalopoulos and Duffy 2016 [45]	Norway	Retrospective cohort	Women aged 50–69 years	500,000	Biennial two-view mammography	1996–2009	13	10,014 screen-detected cancers	16.0
Baines et al., 2016 [46]	Canada	Randomized screening trial	Women aged 40–59 years	89,835	Biennial two-view mammography	1988–2005	17	484 screen-detected cancers	25.0
Falk and Hofvind 2016 [47]	United Kingdom	Retrospective cohort	Women aged 50–79 years	100,000	Biennial two-view mammography	2008–2010	2	273 invasive breast cancers	1.9
Seigneurin et al., 2016 [48]	France	Retrospective cohort	Women aged 50–74 years	100,000	Biennial two-view mammography	2007–2010	3	218 invasive breast cancers and 84 in-situ cancers	17.0
Jorgensen et al., 2017 [49]	Denmark	Retrospective cohort	Women aged 40–84 years	94,932	Biennial two-view mammography	1980–2010	30	271 invasive breast cancer tumors and 179 ductal carcinomas in situ	24.4
van Luijt et al., 2017 [50]	Norway	Retrospective cohort	Women aged 50–70 years	100,000	Biennial two-view mammography	1970–2009	39	324 breast cancer cases	3.0
Autier et al., 2017 [51]	Netherlands	Retrospective cohort	Women aged 50–75 years	100,000	Biennial two-view mammography	1989–2012	23	585 invasive breast cancers and 47 in-situ cancers	52.0
Harding et al., 2019 [52]	United States	Retrospective cohort	Women aged ≥ 40 years	645,057	Biennial two-view mammography	1996–2009	13	104,000 breast cancer cases	31.0
Fann et al., 2019 [53]	Sweden	Retrospective cohort	Women aged 50–70 years	1346	Biennial two-view mammography	1996–1998 2006–2010	15	NM	11.8
Chootipongchaivat et al., 2020 [54]	United States	Retrospective cohort	Women aged ages 30–79 years	100,000	Biennial two-view mammography	1975–2015	40	NM	6.5
Blyuss et al., 2021 [55]	United Kingdom	Case-control	Women aged 47–89 years	163,146	Biennial two-view mammography	2010–2011	2	NM	9.5
Ryser et al., 2022 [56]	United States	Retrospective cohort	Women aged 50–74 years	35,986	Biennial two-view mammography	2000–2018	18	718 breast cancer cases	15.4
Ding et al., 2022 [57]	Belgium	Retrospective cohort	Women aged 50–69 years	100,000	Biennial two-view mammography	2001–2011	10	18 invasive breast cancers	20.1

NM, not mentioned.

**Table 2:** Effect analysis of included articles.

Model	Effect Size and 95% Interval				Prediction Interval		Between-Study		Other Heterogeneity Statistics			
	Number Studies	Point Estimate	Lower Limit	Upper Limit	Lower Limit	Upper Limit	Tau	TauSq	Q-Value	df (Q)	*p*-Value	I-Squared
Fixed	30	0.177	0.177	0.177	0.033	0.379	0.693	0.480	413,510.290	29	0.000	99.993
Random	30	0.126	0.101	0.156								

**Table 3:** Classic and Orwin’s fail-safe N outcomes.

Classic Fail-Safe N Method		Orwin’s Fail-Safe N Method	
Z-value for observed studies	1200.30110	The event rate observed in studies	0.17712
*p*-value for observed studies	0.00000	The criterion for a “trivial” event rate	0.50000
Alpha	0.05000	Mean event rate in missing studies	0.50000
Tails	2.00000	Number of missing studies that would bring the *p*-value to > alpha (N-value)	The criterion must fain between other values
Z for alphas	1.95996		
Number of observed studies	30.00000		
Number of missing studies that would bring the *p*-value to >alpha (N-value)	1342.00000		

**Table 4:** Kendall’s tau with/without continuity correction and Egger’s regression intercept.

Kendall’s S Statistic (P-Q)	−45.00000
**Kendall’s tau with continuity correction**	
Tau	−0.10345
Z-value for tau	0.80285
*p*-value (1-tailed)	0.21103
*p*-value (2-tailed)	0.42206
**Kendall’s tau without continuity correction**	
Tau	−0.10115
Z-value for tau	0.78501
*p*-value (1-tailed)	0.21623
*p*-value (2-tailed)	0.43245
**Egger’s regression intercept**	
Intercept	−27.36434
Standard error	33.06605
95% low limit (2-tailed)	−95.09708
95% upper limit (2-tailed)	40.436839
t-value	0.82757
df	28.00000
*p*-value (1-tailed)	0.20746
*p*-value (2-tailed)	0.41491

## Data Availability

Not applicable.

## Supplementary Materials

The following supporting information can be downloaded at: https://www.mdpi.com/article/10.3390/jpm13030523/s1, Table S1: PRISMA recommendations checklist [74]; Table S2: PubMed search strategy.
